# Editorial: Affect in Sports, Physical Activity and Physical Education

**DOI:** 10.3389/fpsyg.2021.785814

**Published:** 2021-11-11

**Authors:** Darko Jekauc, Claudio R. Nigg, Martina Kanning, David M. Williams, Ingo Wagner, Ryan E. Rhodes

**Affiliations:** ^1^Department for Health Education and Sport Psychology, Institute for Sport and Sport Science, Karlsruhe Institute of Technology, Karlsruhe, Germany; ^2^Department of Health Science, Institute of Sport Science, University of Bern, Bern, Switzerland; ^3^Department of Health and Social Sciences in Sport Science, University of Konstanz, Konstanz, Germany; ^4^Department of Behavioral and Social Sciences, Brown University School of Public Health, Providence, RI, United States; ^5^Department for Interdisciplinary Science, Technology, Engineering and Mathematics Education and Physical Education, Institute for Sport and Sport Science, Karlsruhe Institute of Technology, Karlsruhe, Germany; ^6^School of Exercise Science, Physical and Health Education, University of Victoria, Victoria, BC, Canada

**Keywords:** affective states, emotion, mood, exercise, physical activity, physical education

Affect is a central theme of scientific inquiry in psychology, since its earliest debut. In recent years, substantial progress has been made to deepen our knowledge of how affective states (i.e., core affect, mood) and emotions influence our lives (Williams et al., 2018; Jekauc et al., 2019; Giurgiu et al., 2020). As sports, exercise, and physical activity represent behaviors with considerable properties related to affect, this additional knowledge across several scientific disciplines has the potential to stimulate research. Currently, emotions and affective states represent an important topic in sport and exercise psychology that has gained significant prominence in recent years. It has been speculated that there are three areas of application in the context of sport, exercise, and educational psychology.

First, the results of several studies suggest that affective constructs have a significant impact on motivation to adopt a physically active lifestyle (Jekauc and Brand, 2017). Accordingly, evidence seems to support a hypothesis showing that affective constructs are consistently related to physical activity (Rhodes et al., 2009; Rhodes and Kates, 2015). Second, emotions are supposed to have a considerable influence on performance in competitive sports (Kopp and Jekauc, 2018; Fritsch and Jekauc, 2020). For example, there is ample evidence that certain emotions, such as anxiety, can affect performance in important competitions (Woodman and Hardy, 2003), although the mechanisms of action are still unclear. Third, learning processes in physical education are also thought to be influenced by affective phenomena (Simonton et al., 2021). However, this last area of research is less well-explored.

The aim of this Research Topic was to investigate the relationship between affective processes and action in these three areas of application. In the context of this Research Topic, it became clear that the topic of emotions and affective processes plays a much greater role in research in competitive sports and exercise than in physical education. Eight papers in this Research Topic addressed aspects of exercise and physical activity, six papers addressed aspects of competitive sports, and unfortunately, no paper addressed aspects of physical education.

## Exercise Psychology

In the field of exercise psychology, affective processes are considered as determinants of physical activity. Depending on which paradigm a study is based on, affective processes play different roles and are assigned to different constructs. While in the context of the social cognitive approach affective processes are attributed to cognitive constructs such as affective attitudes or affective outcome expectancies (French et al., 2005; Conner et al., 2011), in the context of dual process theories affective processes are understood as determinants of the implicit processes of behavior control (Williams and Bohlen, 2019; Strobach et al., 2020). Several dozen affective constructs now exist that can be considered as determinants of physical activity. Stevens et al. attempted to bring some clarity and structure to the discussion of affective determinants in their narrative review. As the final product of this review, the authors adapt the Affect and Health Behavior Framework (Williams and Evans, 2014) for exercise behavior, with four main categories of affective constructs that aim to provide an organizing structure in which the many different affects and affect-related constructs in sport psychology can be located. This raises the question of whether affective constructs can be influenced as part of interventions to increase levels of physical activity. In a meta-analysis, Chen et al. found that affective constructs can indeed be effectively influenced by interventions to increase physical activity levels. Positive affective variables partially mediated intervention effects on physical activity, and both indirect and direct effects were significant, implying that other non-affective constructs besides affective variables play a role in driving health behaviors.

One such mechanism of how affective variables influence physical activity is habit formation. In their longitudinal study with many measurement time points, Weyland et al. found that positive affective states during exercise are significantly involved in the development of physical activity habits. Therefore, one strategy to promote health-related habits might be to promote positive affective states during exercise. Slawinska and Davis suggest that in addition to the actual affect experienced during exercise, recalled affect also has an influence on behavioral control, with the intensity of exercise and time playing a role in this process. In this context, Box and Petruzzello note that high-intensity interval exercise is one of the top trends in the fitness industry and this popularity is likely due to the affective processes during high-intensity exercise. Another potential affective determinant of physical activity in young adults is anticipated shame, which, however, was found to be an inconsistent predictor in Garn and Simonton's study. To explain motivation for physical activity and sedentary behavior, Stults-Kolehmainen et al. developed the WANT model (Wants and Aversions for Neuromuscular Tasks) based on findings from neurophysiological research, which postulates two unrelated dimensions (wants vs. aversion). To test the assumptions of this model, Stults-Kolehmainen et al.'s developed a questionnaire to measure these two dimensions as part of a second paper in this Research Topic. The results of psychometric analyses seem to confirm the assumptions of the WANT model and suggest good psychometric properties.

## Sport Psychology

In the field of sport psychology, emotions can be considered as determinants of athletic performance. However, until now, clear evidence based on experimental research has been lacking. To address this research gap, Giles et al. addressed the research question of the extent to which approach-oriented emotions (i.e., anger) and avoidance-oriented emotions (e.g., fear) could be systematically manipulated by an intervention and the extent to which runners' performances could be influenced by the induced emotions. The results of the study confirmed the hypothesis that approach-related emotions could enhance runners' performance. In another study, Fritsch et al. investigated the situational factors influencing emotional expressive behavior in real table tennis matches. The results showed that the importance of the situation (e.g., big points) consistently influenced the expression of positive and negative emotions, whereas the controllability of the situation did not always influence the athletes' expressive behavior. In addition, the results of this study could not confirm the hypothesis that emotional expression behavior influences the probability of winning and losing the next point. In another study, Dong et al. found that systematic use of probiotics, such as *Bifidobacterium animalis* subsp. lactic BB-12, could significantly reduce cognitive and somatic anxiety and led to increased performance in young divers.

In addition to the question of how emotions arise in sport and what consequences they have for athletes' performance, it is very significant to investigate how emotions can be effectively regulated. One strategy of emotion regulation is self-distancing, which was shown to be an effective strategy for reducing aggressive behavior and negative affect in the study by Michel-Kröhler et al. Two other studies examined how specific emotions could be measured in the context of competitive sport. Rice et al. examined the psychometric properties of the Athletic Perceptions of Performance Scale when assessing athlete-specific guilt and shame in junior elite cricketers. The analyses largely confirmed that shame-proneness mediated the relationship between general and athlete-specific distress, whereas guilt-proneness was not a significant mediator. Finally, Han et al. examined the extent to which the mood profiles identified in many English-language studies could also be found in other cultural communities. The results of a study with participants from Singapore could confirm these six hypothesized mood profiles.

## Conclusion

In sport and exercise psychology, there is a growing awareness that emotions influence multiple processes such as motivation, performance, or learning, and thus have a significant impact on people's actions in a sport context. The contributions cover a wide range of exciting new questions, spanning from the emergence of emotions in competitive sports to the effects of affective states on habit formation. However, research in the area of physical education is comparatively scant and not represented at all within the scope of this Research Topic. Obviously, our Research Topic has not reached researchers in physical education and possibly such research is difficult and faces many formal obstacles in the context of schools. Notwithstanding these difficulties, research in the context of school sports is crucial to our understanding of emotional processes.

It is noteworthy that a variety of methods have been used in the contributions to this Research Topic, including correlational, longitudinal, and experimental designs that include behavioral and self-report measures. To reach the next level in the study of affective processes in the context of sport, it will probably be important to also include objective methods of affect capture such as skin conductance, heart rate, or hormone concentration in saliva. Encouragingly, we found that one meta-analysis, one narrative review, and three papers on the development of new measurement instruments to assess emotion-related constructs were authored. We see in these endeavors that new and innovative approaches to the study of emotional processes in the context of sport are being taken.

## Author Contributions

All authors listed have made a substantial, direct and intellectual contribution to the work, and approved it for publication.

## Conflict of Interest

The authors declare that the research was conducted in the absence of any commercial or financial relationships that could be construed as a potential conflict of interest.

## Publisher's Note

All claims expressed in this article are solely those of the authors and do not necessarily represent those of their affiliated organizations, or those of the publisher, the editors and the reviewers. Any product that may be evaluated in this article, or claim that may be made by its manufacturer, is not guaranteed or endorsed by the publisher.
